# VO_2_(B)/Graphene Composite-Based Symmetrical Supercapacitor Electrode via Screen Printing for Intelligent Packaging

**DOI:** 10.3390/nano8121020

**Published:** 2018-12-07

**Authors:** Jieyu Zhang, Liangzhe Chen, Yixiang Wang, Shaoyong Cai, Huijun Yang, Hao Yu, Fuyuan Ding, Chi Huang, Xinghai Liu

**Affiliations:** 1 School of Printing and Packaging, Wuhan University, No. 299, Av. Bayi, Wuhan 430072, Hubei, China; jyzhang0404@whu.edu.cn (J.Z.); chen_lz1991@whu.edu.cn (L.C.); 2017202170003@whu.edu.cn (S.C.); 2018202170010@whu.edu.cn (H.Y.); yuhao1979@hotmail.com (H.Y.); dingfuyuan@whu.edu.cn (F.D.); 2 Department of Food Science and Agriculture Chemistry, McGill University, 21111 Lakeshore, Ste Anne de Bellevue, Quebec, QC H9X3V9, Canada; yixiang.wang@mcgill.ca; 3 College of Chemistry and Molecular Sciences, Wuhan University, No. 299, Av. Bayi, Wuhan 430072, Hubei, China; chihuang@whu.edu.cn

**Keywords:** VO_2_(B)/GN core-shell composites, screen printing, symmetrical supercapacitor, flexible energy storage devices, intelligent packaging

## Abstract

More multipurpose and convenient demand driven by Radio Frequency Identification (RFID) and intelligent packaging require flexible power sources. A VO_2_(B)/graphene (VO_2_(B)/GN) core-shell composite was successfully synthesized by the hydrothermal treatment with V_2_O_5_ and graphite. The as-obtained sample was characterized by XRD, FT-IR, SEM, TEM, and XPS measurements. In addition, the electrochemical properties of VO_2_(B)/GN were tested. Due to its great electrochemical performance and mechanical properties, graphene could increase the electrochemical performance and strengthen the structural stability of the material at the same time. With increasing loading amount of GN, the specific capacitance of VO_2_(B)/GN increased correspondingly. With 20% GN loading, the initial discharge specific capacity could reach 197 F g^−1^ at 0.5 A g^−1^, and 160 F g^−1^ at 1 A g^−1^ in 0.5 M Na_2_SO_4_ electrolyte, which is better than that of pure rod-like VO_2_(B). The capacitance of the VO_2_(B)/GN (20%) composite electrode retains 95.49% after 1000 cycles, which is higher than that of a pure VO_2_(B) electrode (85.43%), indicating that the VO_2_(B)/GN composite possesses better cycling stability. Moreover, a symmetrical solid-state supercapacitor (SCs) using VO_2_(B)/GN(20%) as the anode was assembled. Four printed SCs were connected in series to light up a 1.5 V red LED. This demonstrates its potential application in intelligent packaging to trace food safety.

## 1. Introduction

Food indicators applied in intelligent packaging have aroused worldwide attention. Furthermore, the use of power-driven indicators has been an emerging trend to set up the interaction between customers and products by displaying product information and freshness. Among them, flexible supercapacitors become a promising candidate. The rapid growth of flexible and portable electronics has stimulated enormous efforts to develop multi-purpose, high-power supercapacitors [1,2,3,4,5,6]. Nowadays, flexible supercapacitors are emerging research directions in rechargeable batteries. The development of high-performance electrode materials with certain mechanical flexibility has become one of the frontiers in the field of energy storage [7]. At present, flexible batteries are mainly used in scroll screens, wearable electronic devices, electronic smart cards, and implantable medical devices, as well as RFID tags and intelligent packaging [8,9,10,11]. Many research efforts have been done in this emerging field. M.P. Down et al. [12] manufactured paper-based supercapacitors (P-SCs) with acceptable electrochemistry properties using commercially available pencils to draw upon printing paper. However, the reported electrode materials for flexible batteries are still far from practical applications and industrialization. So far, the studies of fabricating SCs through printing becomes a candidate for realizing large scale production of flexible electronics at low cost and large scale. Among various printing technologies, screen printing process is simple, efficient and low-cost [4]. An electrode ink of excellent shear thinning behavior and processability was prepared by Zhang et al. [1] which was well-adapted to all kinds of substrates (paper, textile, PET etc.). Additionally, all-printed solid-state micro-supercapacitors were printed using 3D printing techniques. Foster et al. [13] demonstrated proof-of-concept for the first time and fabricated a range of 3D electrodes via 3D printer utilizing graphene-based polylactic acid (PLA) filament, which could be applied as lithium-ion anodes and SCs. Printed power sources can be easily fabricated on a large scale. Tehrani and his fellows [4] assembled an ultra-thin flexible supercapacitor via screen printing with PET acting as the substrate. The electrode material, current collector and activated carbon layer were all printed, which was promising for cost-effective, high-performance, and mass production for flexible energy storage applications.

Metallic vanadium (V) is a single crystal polyvalent metal [14]. It reacts easily with oxygen to form oxides of various phases and morphologies. Its common systems are as follows: (a) the conversion between the three stable valence states of +2, +4, and +5; (b) the formation of VOx with different valence states of vanadium. Vanadium oxide is a promising electrode material for batteries because of its special lamellar structure (allowing some ions and groups to insert). It has broad application prospects in intelligent packaging and other fields, which has aroused wide attention and a great number of research has been done [15]. The most important application of vanadium oxide is the research of electrode materials. As vanadium is a relatively active transition metal, it can form vanadium oxides with different ligands. It also allows a large number of different groups or metal ions to intercalate reversibly with the typical layered materials. This special crystal structure makes vanadium oxide nanomaterials become a potential candidate for electrode materials [16,17,18,19,20].

Vanadium oxides can be intercalated by lithium (sodium) ions under electrochemical action to form layered LixVO_2_, NaxVO_2_, and other lithium or sodium intercalated complexes. In addition, inorganic metal oxides are often used as electrode materials. However, some irreversible changes in volume often take place when ions insert and extract, and the structures are, therefore, damaged, resulting in poor cyclic stability of the electrode materials. Vanadium dioxides/GN composites could be a great candidate to solve this problem during the application of electrode materials, which would not only improve the cyclic stability of the materials, but also help each component to play a synergistic role in supercapacitors. Deng et al. [21] prepared the graphene/VO_2_ composites materials by a one-step hydrothermal reaction. The RG/VO_2_ electrode material with RG content of 26 wt% can reach 225 F g^−1^ at a current density of 0.25 A g^−1^ in 0.5 mol L^−1^ K_2_SO_4_ solution. A coaxial-structured VO_2_(B)/MWCNT (multiwalled carbon nanotubes) hybrid material was prepared through the sol-gel method by Liang et al. [22], and its specific capacitance can be obtained at 250 F g^−1^ in 1M Na_2_SO_4_ electrolyte at 0.5 A g^−1^. Pan et al. [23] prepared graphene-bridged V_2_O_3_/VO_x_ core–shell nanomaterials, and the assembled electrochemical supercapacitors (ECs) possesses excellent power density of 1000 kW kg^−1^ at an energy density of 10 Wh kg^−1^. Xiao et al. [24] prepared VO_2_(B)/grapheme through a hydrothermal reaction. The initial specific capacitance of the composites reached 290.4 F g^−1^ at 0.2 A g^−1^ in a voltage window from −0.4 to 0.6 V. However, the supercapacitor was not further fabricated to test its electrochemical performance.

**Scheme 1:**
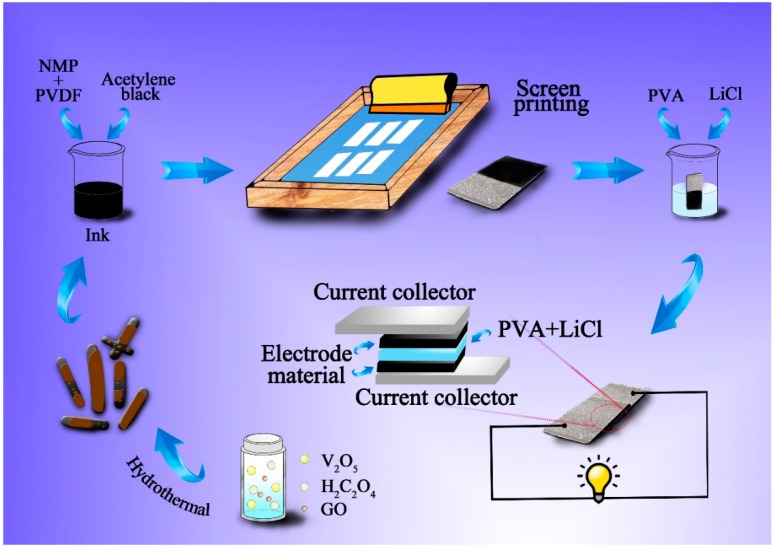
Illustration of the preparation process of VO_2_(B)/GN composites and fabrication procedure of symmetric SC devices.

The as-prepared flexible SCs in this paper can be employed as power source supplying for electronic devices in intelligent packaging, such as LEDs, RFID, electronic sensors, intelligent voice devices, and so on, to trace food information and indicate food freshness. Normally, the power source applied in packaging does not require high voltage and specific capacitance, and studies focus on the properties of flexibility, low-cost, and large-scale production. Herein, in this work, we prepared rod-like VO_2_(B)/GN core-shell composites by a one-pot hydrothermal reaction through mesh printing, which could meet the requirements of power supply for packaging. The electrochemical properties of the VO_2_(B)/GN composite can be optimized by adjusting the mass ratio of GN. Subsequently, its morphology, crystalline structure, chemical bonds, and chemical components were investigated. The whole procedure of preparing VO_2_(B)/GN composites and fabrication of symmetrical supercapacitors are demonstrated in Scheme 1. The as-prepared ink was printed on the Ni foam substrate by screen-printing to fabricate solid-state SCs device, indicating its potential application in volume production. Finally, a red light emitting diode (LED, 1.5 V) was lit up by four assembled SCs devices in series, showing a promising application in intelligent packaging to drive luminous or sounding devices.

## 2. Experimental Details

### 2.1. Materials and Chemicals

Polyvinyl alcohol, *N*-methyl pyrrolidone (NMP), sulfuric acid, phosphoric acid, hydrochloric acid, potassium permanganate, oxalic acid dehydrate, and absolute ethyl alcohol were purchased from Sinopharm Chemical Reagent Co, Ltd. (Shanghai, China), polyvinylidene fluoride (PVDF) was purchased from Arkema Innovative Chemistry Co. Ltd. (Paris, France), vanadium pentoxide was purchased from Sun Chemical Technology Co. Ltd. (Shanghai, China). Acetylene black was purchased from Tianjin Ebory Chemical Co., Ltd. (Tianjin, China). Graphite was purchased from Sigma-Aldrich Co. Ltd. (St. Louis, MO, USA). Lithium chloride (LiCl) was purchased Tianjin Fuchen Chemical Reagents Factory (Tianjin, China). The deionized water used in the experiments was lab-made. All chemical reagents were analytical grade and used without further purification.

### 2.2. Synthesis of Graphite Oxide

The synthetic procedure of graphite oxide is based on the modified Hummers method reported by Marcanos et al. [25]. Firstly, 2.25 g of 325 mesh graphite powder was added into the mixed solution containing H_2_SO_4_/H_3_PO_4_ (volume ratio was 9:1), and 13.5 g of KMnO_4_ was slowly stirred into the mixed solution. Then the mixed solution was heated to 55 °C for 12 h. When the mixture was cooled to room temperature, it was slowly poured into an ice bath containing 2.5 mL 30% H_2_O_2_ until the solution turned to golden yellow and no bubbles were produced. Next, the mixture solution was filtered through 0.45 μm PTFE membrane and washed with 5% HCl and deionized water until it is neutral and without residual SO_4_^2−^ (detected with BaCl_2_). The graphite oxide (GO) was prepared by drying in vacuum at 60 °C for 48 h.

### 2.3. Synthesis of Pure VO_2_(B) Nanorods

To simplify, 0.91 g of V_2_O_5_ powder and 1.26 g of oxalate dihydrate were dissolved in 80 mL deionized water by ultrasonic for 20 min. The mixture was then transferred to stainless steel autoclave and heated at 210 °C for 24 h. The obtained black products was collected and washed by distilled water and absolute ethanol for several times, respectively. The final product was dried in vacuum at 80 °C for 10 h.

### 2.4. Synthesis of VO_2_(B)/GN Composites

Similarly, 0.91 g of V_2_O_5_ powder and 1.26 g of oxalate dihydrate were dissolved in 30 mL deionized water by ultrasonic for 20 min, and a certain amount of GO was dispersed in 30 mL deionized water by ultrasonic. The two above mixtures were then transferred to a stainless steel autoclave and heated at 210 °C for 24 h. The subsequent procedures were the same as those described above and the VO_2_(B)/GN composite was obtained.

### 2.5. Preparation of the VO_2_(B)/GN Ink

Briefly, the VO_2_(B)/GN composites screen printing ink was prepared containing 80 wt% VO_2_(B)/GN composites, 10 wt% acetylene black and 10 wt% polyvinylidene fluoride (PVDF). PVDF was dissolved completely in N-Methyl pyrrolidone (NMP) ultrasonically in advance. Then VO_2_(B)/GN composites and acetylene black were added under continuous stirring until a homogenous ink was formed.

### 2.6. Fabrication of Solid-State Screen Printed Symmetric SCs

Firstly, electrodes were printed by screen printing on Ni foams using as-prepared VO_2_(B)/GN ink, followed by vacuum drying at 60 °C. Screen printing was operated at room temperature using a mesh (100 mesh-counts). Then, the solid-state supercapacitor was assembled from two identical electrodes, where LiCl/polyvinyl alcohol (PVA) gel was used as the electrolyte. For preparing the LiCl/PVA gel, 2 g of PVA and 2 g of LiCl were added simultaneously into deionized water and stirred at 85 °C until a clear gel electrolyte was formed, followed by immersing in the as-prepared electrolyte for 5 min before fabrication. Next, the electrodes were dried at room temperature for 3 h to remove the excessive water. Finally the electrodes were pressed together under a pressure of 1 MPa for 10 min and sealed with plastic wrap to assemble the symmetric supercapacitor. The average mass loading of electrode materials in one supercapacitor was about 6.0 mg.

### 2.7. Characterizations

Scanning electron microscope (SEM, Hitachi S-4800, Hitachi, Tokyo, Japan) images, energy dispersive X-ray spectroscopy (EDX, NanoSEM400 with EDAX Phoenix, Hillsboro, OR, USA) data, and transmission electron microscope (TEM and HRTEM, FEI Tecnai G20, Hillsboro, OR, USA) images were performed to investigate the morphology of VO_2_(B)/GN composites. The X-ray diffraction (XRD, Bruker B8 ADVANCE, Karlsruhe, Germany) from PANalytical X’Pert Pro with Cu Ka radiation and X-ray photoelectron spectroscopy (XPS, Thermo Scientific, Waltham, MA, USA) from Thermo Scientific ESCALAB 250Xi system with Al-Ka as the radiation source to characterize the structural information and phase purity. Raman spectroscopy was characterized on a Confocal Raman Microspectroscopy (Renishaw, RM-1000, Gloucestershire, UK) at room temperature with Ar^+^ laser of 514.5 nm excitation. The chemical bonds information was measured with a Nicolet 5700 Fourier transformed infrared spectrometer (FT-IR, Thermo Scientific, Waltham, MA, USA). The electrochemical activity of the electrodes and SC devices were investigated by using a CS 350 electrochemical workstation in a conventional three-electrode system (Wuhan Corrtest Instruments Corp., Ltd., Wuhan, China).

### 2.8. Electrochemical Study of Electrodes

The VO_2_(B)/GN composites screen printed on Ni foams (0.5 mm thick and purity >99.9%) can be directly used as working electrode where Ni foams acted as the current collector. The Ni foams were sonicated in acetone for 10 min, and washed copiously with 0.1 M HCl, ethanol and deionized water. Then 80 wt% active materials, 10 wt% acetylene black, 10 wt% PVDF, and a few drops of NMP were mixed to form a syrup. It was screen printed on Ni foam and pressed under 10 MPa for 1 min to form the electrode (the thickness is about 0.15 mm), and dried at 60 °C in vacuum overnight. The mass of the active material on each electrode is 3~5 mg. A platinum electrode, a saturated calomel electrode (SCE) and 0.5 M Na_2_SO_4_ solution were used as counter electrode, reference electrode and electrolyte, respectively.

## 3. Results and Discussion

**Figure 1:**
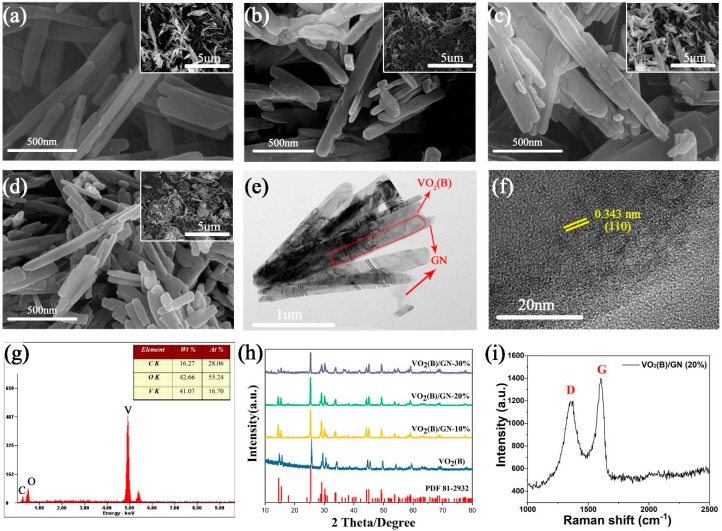
SEM images of (**a**) VO_2_(B), and VO_2_(B)/GN composites with different mass ratio: (**b**) 10%, (**c**) 20%, (**d**) 30%, (**e**) TEM image and (**f**) HRTEM images of VO_2_(B)/GN composites, (**g**) EDX image of VO_2_(B)/GN composites, (**h**) XRD patterns of VO_2_(B) and VO_2_(B)/GN composites, and (**i**) Raman spectrum of VO_2_(B)/GN (20%).

The morphology and particle size of the prepared nanocomposite were characterized by SEM and TEM. As depicted in Figure 1a, VO_2_(B) exist of nanorods. Besides, the as-prepared nanorods are several micrometers long with widths of 40~60 nm. Figure 1b–d show VO_2_(B)/GN composites with different mass ratios, at 10%, 20% and 30%, respectively. With increasing mass ratio of GO, the surface of VO_2_(B) nanorods become more irregular, indicating that adding a certain amount of GO may influence the morphology of VO_2_(B).

This speculation was confirmed by TEM testing. The TEM image of VO_2_(B)/GN (20%) composites are shown in Figure 1e. It is clear that some GN and nanorods can be found together as composites, where GN covers VO_2_ as nets. In the formed core-shell structure, the core is VO_2_ and the shell is GN. The similar structure was reported by Zhang [20,26,27]. Figure 1f shows the HRTEM image of VO_2_(B)/GN composites. The distance between the lattice planes is about 0.332 nm, which is consistent with the (110) lattice plane of VO_2_(B) [28]. Furthermore, the energy dispersive X-ray (EDX) analyses provided additional insight into the chemical composition and content of the surface of VO_2_(B)/GN composites (Figure 1f). The result confirms the presence of vanadium(V) and carbon(C), the ratio of which is 41.07 wt% and 16.27 wt%, respectively. The calculated weight ratio of vanadium and carbon (2.56) is lower than the theoretical value (3.06), which is due to undecomposed oxalic acid or it is not washed thoroughly.

As shown in Figure 1h, the crystalline structure of VO_2_(B) and VO_2_(B)/GN core-shell composite was also characterized by XRD. In VO_2_(B) pattern, the distinguish diffraction peaks can be attributed as monoclinic VO_2_ (space group C2/m) with lattice parameters a = 12.09, b = 3.7021, c = 6.433 Å, which is in line with the VO_2_ (B) (JCPDS card no. 81-2392, a = 12.09, b = 3.702, c = 6.433 Å) [20,29] without other peaks, indicating that the as-prepared composite is in typical pure metastable state phase of VO_2_(B). The strong peak situated at 2θ = 25.00° is correspond to diffraction peak of (110) planes, it is the result of preferred orientation growth of VO_2_(B) [21]. The d-spacing of (110) is 0.346 nm, which is similar to the TEM analysis. Additionally, the similar peaks are shown in VO_2_(B)/GN core-shell composites, revealing the successful combination of VO_2_(B) and GN.

The Raman spectrum of VO_2_(B)/GN (20%) is depicted in Figure 1i. There are two prominent bands at about 1375 cm^−1^ (D band) and 1604 cm^−1^ (G band). The G band corresponds to the vibration in all sp^2^-bonded carbon atoms while the D band can be attributed to the defects of the sample [27]. The sample has an I_D_/I_G_ ratio of 0.86, which is lower than the report for rGO (I_D_/I_G_ > 1) [30]. However, It can be comparable to the value of I_D_/I_G_ for the graphitized graphene (I_D_/I_G_ = 0.83), indicating the existence of some structural defects [31]. Moreover, this conclusion reveals that GO is reduced to GN by oxalic acid, which is in line with the observation in TEM images.

**Figure 2:**
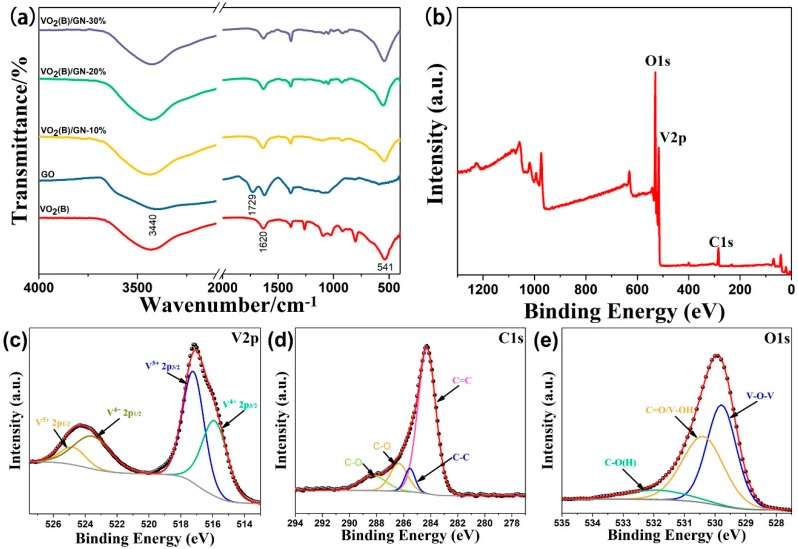
(**a**) FT-IR spectra of VO_2_(B), GO, and VO_2_(B)/GN composite, XPS spectra of VO_2_(B)/GN composite: (**b**) Survey peak; (**c**) typical V 2p; (**d**) typical C 1s and (**e**) typical O 1s.

**Scheme 2:**
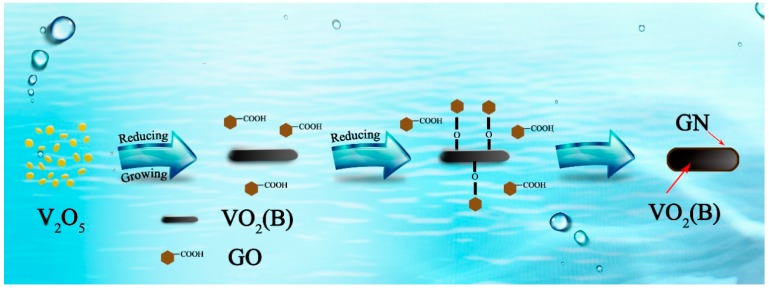
Mechanism of VO_2_(B)/GN composite preparation.

Figure 2a shows the FT-IR spectra of VO_2_(B), GO and VO_2_(B)/GN composite. The broad absorption bands at about 3400 cm^−1^ are assigned to O-H stretching while medium bands at 1620 cm^−1^ are ascribed to bending vibration of H_2_O molecule, which can be observed in all samples. The characteristic bands of VO_2_(B) [32,33], around 541 cm^−1^, related to V–O–V stretching mode of vibration, which are also displayed in VO_2_(B)/GN composite [28,34]. In GO sample, the characteristic band at 1729 cm^−1^ can be assigned to the C=O stretching vibration of carboxyl group (COOH), which disappears in the VO_2_(B)/GN composite, indicating that COOH is reduced by oxalic acid during the hydrothermal reaction. The possible mechanism of VO_2_(B)/GN composite preparation is illustrated in Scheme 2. The V_2_O_5_ particles are reduced and then grow into VO_2_(B) nanorods at relatively high temperature and pressure. The dispersed GO nanosheets with abundant carboxyl groups move over and get reduced on the surface of VO_2_ at the same time. Subsequently, the core-shell structure of VO_2_(B)/GN nanorods are formed.

The proposed mechanism of VO_2_(B)/GN composite preparation are supported by XPS analysis. The survey peak with V, C and O elements is found in Figure 2b and the quantification of percentages of each component is shown in Table 1. As shown in Figure 2c, two oxidation states of vanadium in V 2p spectrum are fitted well with binding energy at 515.9 and 523.6 eV for V^4+^, while the others at 517.2 and 524.8 eV corresponds to V^5+^ [35,36]. The existence of V^5+^ may be due to that the unreduced V_2_O_5_ is absorbed on the surface of VO_2_, which is similar to the assumption reported by Wu and Lian [37]. Figure 2d shows the XPS spectrum of C 1s, the fitted peaks at 284.3, 285.6, 286.3, and 288.1 eV corresponds to C=C, C–C, C–O and COOH groups, respectively [31,38]. In VO_2_(B)/GN, most of COOH groups are reduced by oxalic acid so that it is incapable of being detected by FT-IR. As shown in Figure 2e, the O 1s shows V–O–V (VO_x_), O=C/V–O (H) and O–C peaks at 529.8, 530.4, 531.9 eV, respectively [37,39]. Based on the C 1s and O 1s spectra, certain number of V–O and C–O groups are shown, revealing that the VO_2_(B) and GN are likely to be combined by C–O–V chemical bond.

**Figure 3:**
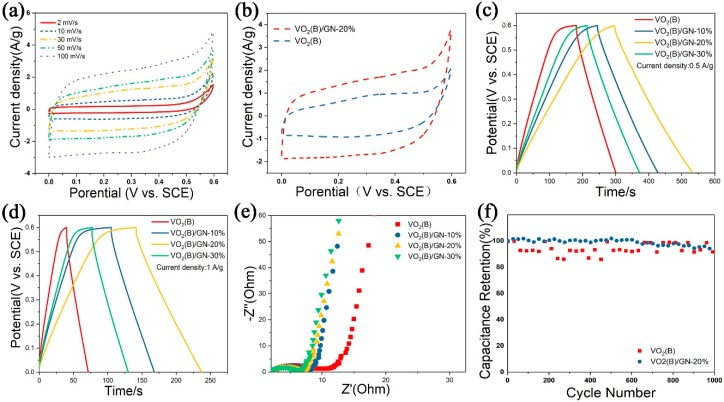
(**a**) The CV curves of pure VO_2_(B)/GN (20%) composite electrodes at different scan rate, (**b**) the CV curves of pure VO_2_(B) and VO_2_(B)/GN (20%) composite electrodes at 50 mV s^−1^, (**c**) the GCD curves of pure VO_2_(B) and VO_2_(B)/GN (20%) composite electrodes at 0.5 A g^−1^, (**d**) the GCD curves of pure VO_2_(B) and VO_2_(B)/GN (20%) composite electrodes at 1 A g^−1^, (**e**) the Nyquist impedance plots of pure VO_2_(B) and VO_2_(B)/GN composites electrodes, and (**f**) the cycling stability of pure VO_2_(B) and VO_2_(B)/GN (20%) composite electrodes at 15 A g^−1^.

Figure 3a displays the cyclic voltammetry (CV) curves of VO_2_(B)/GN electrode between 0 and 0.6 V versus SCE at different scanning rate from 2 mV s^−1^ to 100 mV s^−1^; Figure 3b displays the CV curves of pure VO_2_(B) and VO_2_(B)/GN (20%) at a scanning rate of 50 mV s^−1^. It is40 tested to demonstrate the capacitive performance of the VO_2_(B)/GN composite electrode. In Figure 3a, VO_2_(B)/GN (20%) electrode material shows a rectangular shape in this voltage range. However, there is still a relatively large deformation compared with the standard rectangle, indicating that the electrode material has lower internal resistance. Additionally, with an increasing of scan rate, the peak current of cathode and anode increases, and the potential changes along the positive direction, indicating that Na^+^ has been embedded and detached in the material to maintain electrical neutrality. The positions of redox peaks at different scanning rates remained unchanged, indicating that VO_2_(B) has rather satisfactory cyclic reversibility. Additional, the near-rectangular shapes of the CV curves still can be found at higher scanning rates.

The reaction process of VO_2_(B) nanobelts in these three electrodes system is shown below [20]:

VO_2_ + xNa^+^ + xe^−^ = Na_x_VO_2_

Better capacitive behavior of VO_2_(B)/GN (20%) can be observed form a larger area of CV curve (Figure 3b). The CV curves of VO_2_(B)/GN (20%) composite electrode displays a more regular shape, demonstrating the typical pseudocapacity. Furthermore, there are no obvious redox peaks in curves of VO_2_(B) and VO_2_(B)/GN (20%), which can be rationalized by the mild redox behavior for VO_2_(B) and GN [10,40].

Galvanostatic charge/discharge (GCD) is performed to investigate the rate capability and capacitive performance of VO_2_(B) and VO_2_(B)/GN (20%) composite electrodes. Figure 3c,d show the GCD curves of pure VO_2_(B) and VO_2_(B)/GN (20%) composite electrodes at 0.5 A g^−1^ and 1 A g^−1^, respectively. Excellent capacitive behavior can be observed from their linear curves, indicating that their capacitance mainly comes from double layer capacitance. The specific capacitance (C) of VO_2_(B) and VO_2_(B)/GN (20%) composite electrodes are calculated by the following formula:

(1) $$C=\frac{I\times \Delta t}{m\times \Delta V}$$

where *I* is the applied current (A), Δ*t* is the discharge time (s), *m* is the mass of active material (g), and Δ*V* is the potential window (V). The discharge time of VO_2_(B)/GN (20%) indicates higher specific capacitance of 160 F g^−1^ at 1A g^−1^, in comparison to the lower specific capacitance of 64, 105, 92 F g^−1^ for VO_2_(B), VO_2_(B)/GN (10%) and VO_2_(B)/GN (30%), respectively. Additionally, the specific capacitance of the VO_2_(B)/GN (20%) composite electrode is about 197 F g^−1^ at 0.5 A g^−1^, which performs better than 99, 153, and 134 F g^−1^ of VO_2_(B), VO_2_(B)/GN (10%), and VO_2_(B)/GN (30%) electrode materials. The specific capacitance of VO_2_(B)/GN (20%) composite electrode is about 160 F g^−1^ at 1 A g^−1^, which is much higher than 64 F g^−1^ of VO_2_(B) electrode. Furthermore, the specific capacitance of the VO_2_(B)/GN (20%) composite electrode is about 197 F g^−1^ at 0.5 A g^−1^, which performs better than the 99 F g^−1^ of VO_2_(B) electrode. Figure 3f shows the cycling stability of pure VO_2_(B) and VO_2_(B)/GN (20%) composite electrodes at 15 A g^−1^ for 1000 charge/discharge cycles. The specific capacitance of VO_2_(B)/GN (20%) composite electrode retains 95.49% of the original after 1000 cycles, which is higher than that of pure VO_2_(B) electrode (85.43%) and displays better cycling stability.

Figure 3e shows the Nyquist impedance plots of pure VO_2_(B) and VO_2_(B)/GN (20%) composites electrodes to further evaluate their electrochemical performance. The electrochemical impedance spectroscopy (EIS) curves of VO_2_(B) and VO_2_(B)/GN composites consist of a semicircular high frequency region and a straight line low frequency region. The semicircle in the high frequency region mainly comes from the charge transfer reaction at the interface between the electrolyte and active material, which corresponds to the process of electron transfer. The point of contact between the high frequency region and the real axis is considered as the internal resistance of the electrode material. Additionally, the linear type low frequency region is mainly caused by Warburg impedance produced by the diffusion of Na^+^ at the interface of the working electrode, which corresponds to the process of diffusion process [40], indicating that the redox reaction of V(V)/V(IV) is carried out by charge transfer and diffusion simultaneously.

The high frequency semicircle is produced by charge transfer reaction at the electrolyte/oxide electrode interface, corresponding to charge transfer (Rct) [41]. EIS curves show that the internal resistance of VO_2_(B)/GN composites is obviously lower than that of VO_2_(B), showing that the introduction of GN improves the conductivity of the composites. In addition, with increasing mass ratio of GN, the reaction resistance decreases, proving that the addition of GN is beneficial to reduce the reaction resistance of electrode materials. Moreover, the linear slope of the composite is greater than that of VO_2_(B) in the low frequency region, which also demonstrates that the incorporation of GN into the composite can improve the diffusion rate of Na^+^ and is also consistent with the CV curves. The specific morphology and synergistic effect of VO_2_(B)/GN composites facilitate the capacitive performance.

**Figure 4:**
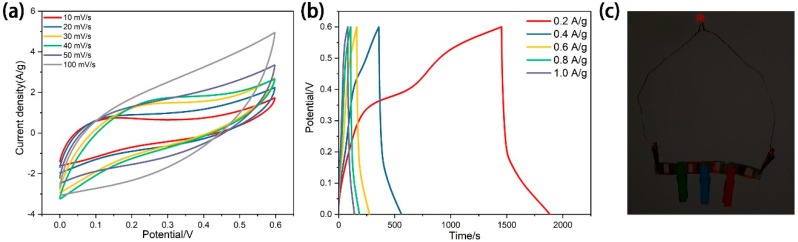
(**a**) The CV curves of screen printed SCs device at 10–100 mV s^−1^, (**b**) the GCD curves of screen printed SCs device at 0.2–1.0 A g^−1^, (**c**) image of four screen-printed SCs connected end to end lighting up a red LED.

To develop flexible power sources, the solid-state flexible SCs based on VO_2_(B)/GN (20%) composite is fabricated via screen printing. Figure 4a displays the CV curves of screen printed SCs device ranging from 10 to 100 mV s^−1^. As shown in Figure 4b, the GCD curves of screen printed SCs device at 0.2–1.0 A g^−1^ are shown. Superb capacitive behavior can be observed from their linear curves. The capacitance (C) can be calculated with Equation (2):

C = (2 × *I* × *t*)/(*m* × ∆*V*) (2)

where *I* is the discharge current (A), *t* is the discharge time (s), *m* is the total mass of one electrode (g), and Δ*V* is the discharge voltage window (*V*). Obviously, with the augment of current densities from 0.2 to 1.0 A g^−1^, the corresponding areal specific capacitance of symmetrical SCs are 287.5, 268.9, 229.3, 209.6, and 196.4 F g^−1^. It exhibits great rate capability.

Moreover, after connecting four symmetrical SCs in series, they can light up a 1.5 V red LED as shown in Figure 4c. Such flexible supercapacitor can be applied in smart packaging to drive LED or other luminous devices to indicate freshness of foods or display product information. It realizes the interaction between customers and information.

## 4. Conclusions

In summary, a series of VO_2_(B)/GN composites were successfully prepared by one-pot hydrothermal reaction. When the mass ratio of GN reaches 20%, optimal electrochemical properties can be obtained. The VO_2_(B)/GN composites displays higher specific capacitance and better stability compared to pure VO_2_(B). The VO_2_(B)/GN (20%) composite specific capacitance of 160 and 197 F g^−1^ at 1 and 0.5 A g^−1^, respectively. It is much higher than that of pure VO_2_(B) (64 and 99 F g^−1^). Additionally, the capacitance value of VO_2_(B)/GN (20%) composite electrode retains 95.49% after 1000 cycles, which is significantly higher than that of pure VO_2_(B) (85.43%) along with a better cycle stability. To develop flexible power sources, the solid-state flexible SCs based on VO_2_(B)/GN (20%) composite is fabricated by screen printing with excellent performance. The assembled symmetric SCs can light up a red LED of 1.5V when four of them are connected in series. More supercapacitor behaviors and its application in intelligent packaging will be investigated in our future work.

## Figures and Tables

**Table 1:** XPS analysis of VO_2_(B)/GN.

Element	Elemental Atom (%)	Moiety		
		Assignment	BE (eV)	Elemental of Signal (%)
V 2p	19.06	V^5+^	517.2, 524.8	47.66
		V^4+^	515.9, 523.6	52.34
C 1s	23.44	C=C	284.3	76.34
		C–C	285.6	12.20
		C–O	286.3	9.81
		COOH	288.1	1.65
O 1s	55.17	V–O–V (VO_x_)	529.8	47.09
		O=C/V–O (H)	530.4	43.50
		O–C	531.9	9.41
